# Seeing the Trees From the Forest: Challenges in Subgroup Analysis-Based Guidelines in Oncology

**DOI:** 10.3389/or.2024.1355256

**Published:** 2024-05-24

**Authors:** Ofer Rotem, Karyn Revital Geiger, Ekaterina Hanovich, Mor Moskovitz, Noga Kurman, Daniel Reinhorn, Idit Peretz, Rinat Yerushalmi, Salomon M. Stemmer

**Affiliations:** ^1^ Davidoff Center, Rabin Medical Center, Petah Tikva, Israel; ^2^ Leumit Health Services, Tel Aviv, Israel; ^3^ Coller School of Management, Tel Aviv University, Tel Aviv, Israel; ^4^ Faculty of Medicine, Tel Aviv University, Tel Aviv, Israel

**Keywords:** cancer, clinical outcomes, clinical trial, guidelines, reporting, subanalysis, treatment

## Abstract

As clinical trials in oncology require substantial efforts, maximizing the insights gained from them by conducting subgroup analyses is often attempted. The goal of these analyses is to identify subgroups of patients who are likely to benefit, as well as the subgroups of patients who are unlikely to benefit from the studied intervention. International guidelines occasionally include or exclude novel medications and technologies for specific subpopulations based on such analyses of pivotal trials without requiring confirmatory trials. This Perspective discusses the importance of providing a complete dataset of clinical information when reporting subgroup analyses and explains why such transparency is key for better clinical interpretation of the results and the appropriate application to clinical care, by providing examples of transparent reporting of clinical studies and examples of incomplete reporting of clinical studies.

## Introduction

Clinical trials require major investment by investigators, sponsors, and participants. Therefore, maximizing the number of insights gained from each trial is often attempted. Specifically, practitioners, researchers, and regulatory agencies alike, seek to identify subgroups of patients who are likely to benefit from the studied intervention, as well as subgroups of patients who are unlikely to benefit from it [1]. Distinguishing between these subgroups is particularly important in the era of personalized medicine. International guidelines occasionally include or exclude novel medications and technologies for specific populations based on subgroup analyses of pivotal trials without confirmatory trials for the subgroup of interest.

A prominent example is the evolution of the studies and indications for epidermal growth factor receptor (EGFR) tyrosine kinase inhibitors (TKIs) in metastatic non-small-cell lung carcinoma (NSCLC), which was based on subgroup analyses. Initially, the findings supported treating all metastatic NSCLC patients with EGFR TKIs, then only the light/never smoker population, and eventually the results supported limiting the use of EGFR TKIs only to those with EGFR mutations. Consequently, the guidelines for EGFR TKIs shifted from EGFR TKI’s as third-line therapy for all patients with advanced NSCLC to first-line therapy for NSCLC patients with EGFR mutations [2–9].

Conducting confirmatory trials for a specific subpopulation is costly and may take several years, while in the meantime, the subpopulation that could have benefited from the intervention is undertreated. Thus, subgroup analysis is a practical approach in the real world. However, forming guidelines and making clinical decisions based on subgroup analyses alone present several major challenges. One of the more commonly discussed challenges is statistical, since the probability of at least one type 1 error (i.e., a false result that falls within statistical significance) increases with the number of tests run on the same data, and the smaller sample size (per subgroup) makes it harder to reach statistical significance. These statistical issues, which have been discussed in many articles, and for which solutions were offered, [1, 10, 11] are beyond the scope of the current article.

This Perspective focuses on the importance of providing a complete dataset of clinical information when reporting subgroup analyses and explains why such transparency is key for interpreting the results of such analyses, and appropriately applying them to clinical care.

## Examples of Transparent Reporting

In 2016, the U.S. Food and Drug Administration (FDA) approved pembrolizumab as first-line monotherapy for patients with metastatic NSCLC with programmed death-ligand 1 (PD-L1)≥50% with no EGFR or anaplastic lymphoma kinase (ALK) genomic tumor aberrations based on the data from the KEYNOTE-024 study. The FDA expanded the indication in 2019 to include metastatic NSCLC with PD-L1≥1% and no EGFR or anaplastic ALK genomic tumor aberrations [12]. This expansion was based on data from the KEYNOTE-042 study, which demonstrated the superiority of first-line pembrolizumab monotherapy over standard-of-care chemotherapy (platinum-based doublet) in patients with PD-L1-positive metastatic NSCLC, in the three pre-planned subgroups (PD-L1>50%, PD-L1>20%, and PD-L1>1%) [13]. However, exploratory subgroup analysis failed to show the benefit of pembrolizumab over chemotherapy alone in patients with PD-L1 1%–49%, and thus, some international guidelines and regulatory authorities including the National Comprehensive Cancer Network® (NCCN®), the American Society of Clinical Oncology (ASCO), and the European Medicines Agency (EMA) still recommend pembrolizumab monotherapy only for patients with PD-L1 expression ≥50% [14–16]. As the KEYNOTE-042 report provided a complete dataset of clinical information, it was possible to confirm that baseline characteristics between the two treatment groups were well-balanced, and that the published progression-free survival (PFS) and overall survival (OS) Kaplan-Meier plots of the placebo groups were consistent with previously published data, thereby supporting the validity of the guideline decisions [13]. Notably, demonstrating that the groups are well balanced with respect to measured baseline characteristics, is necessary but insufficient evidence that the groups are balanced, as unknown/unmeasured confounders may still be unbalanced (as the randomization is broken).

The importance of transparent reporting for subgroup analysis-based guidelines is further illustrated by the PACIFIC trial, which demonstrated superior PFS and OS (two coprimary endpoints) for durvalumab vs. placebo in patients with stage III unresectable NSCLC following definitive concurrent chemoradiotherapy (cCRT) (PFS hazard ratio [HR] 0.52, 95% confidence interval [CI] 0.42–0.65; OS HR, 0.68, 99.73% CI 0.47–0.997) [17, 18]. Based on these results, the FDA approved this drug, and the NCCN guidelines recommended durvalumab for all patients [19, 20]. In contrast, the EMA and the European Society for Medical Oncology (ESMO) recommended the addition of durvalumab only for patients with PD-L1≥1% based on post-hoc subgroup analysis, which demonstrated an OS HR of 1.14 (95% CI, 0.71–1.84) and PFS HR of 0.73 (95% CI, 0.48–1.11) in PD-L1-negative patients [21–23]. Importantly, evaluation of PD-L1 levels was not obligatory in this trial. Determining PD-L1 levels in pre-cCRT archival tumor samples was optional and available for 63% of patients. Of these, the expression levels were retrospectively reported according to prespecified and post-hoc tumor cell cutoffs (25% and 1%, respectively). Additionally, primary endpoints were not defined or stratified by PD-L1 levels. However, similar to the reporting on the KEYNOTE-042 trial, the reports on the PACIFIC trial included all patient characteristics and PFS as well as OS Kaplan-Meier plots, thereby facilitating a clinically meaningful interpretation of the data and helping clinicians decide which guidelines to follow [23–26].

**FIGURE 1:**
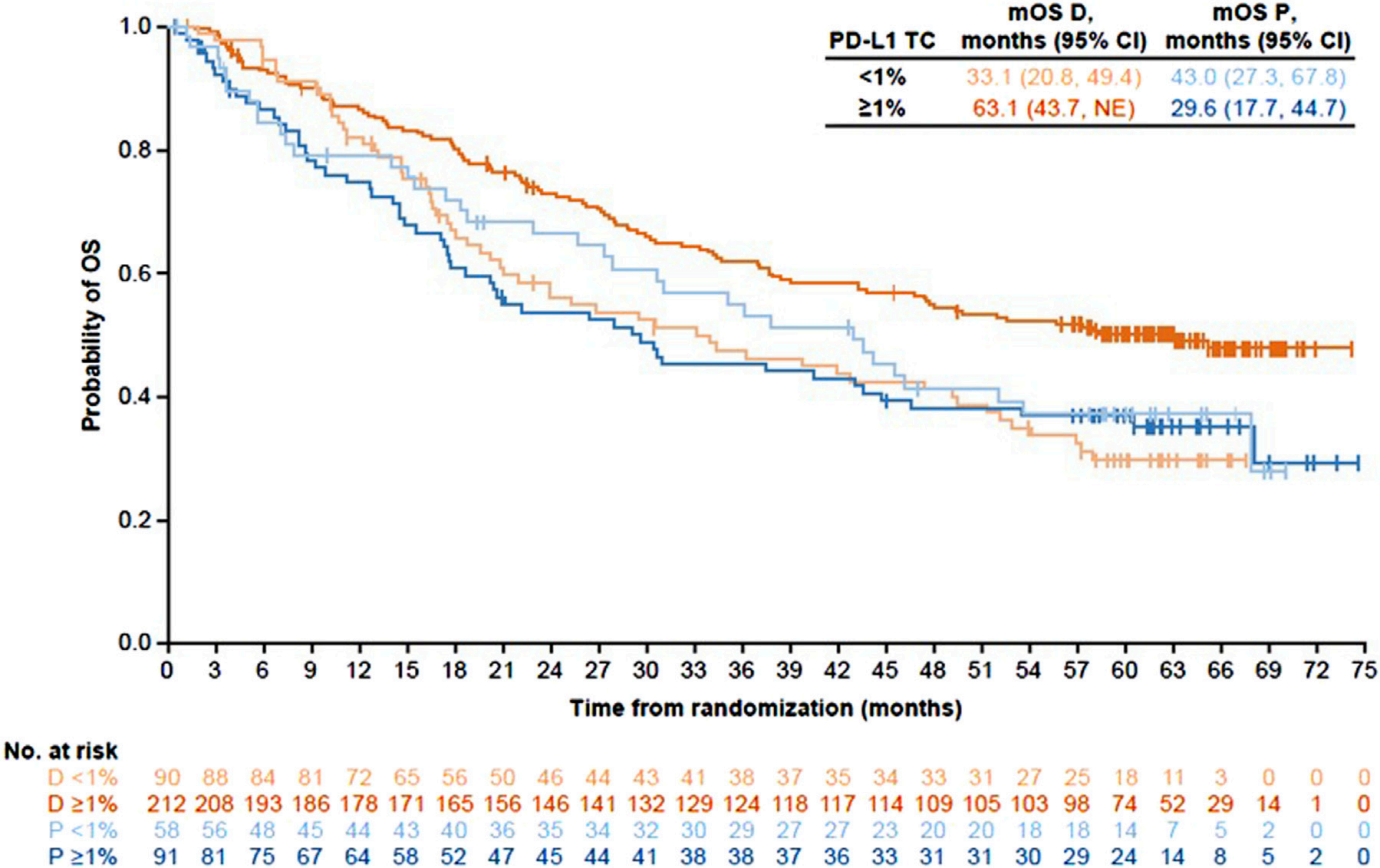
Kaplan-Meier plots for overall survival comparing treatment with durvalumab to treatment with placebo by PD-L1 expression in the PACIFIC trial. Produced from published data [24].

The information provided in the EMA package insert was consistent with the post-hoc subgroup analysis and showed improved clinical outcomes with increased PD-L1 levels in the durvalumab but not the placebo arm [21]. However, further scrutiny of the reported data undermines the validity of the recommendation *not* to treat PD-L1-negative patients. Specifically, comparing baseline characteristics of PD-L1-negative patients between the two treatment arms revealed an imbalance in favor of the placebo arm. PD-L1-negative patients in the placebo arm were more likely to be younger (<65 years), have nonsquamous histology, or have a stage IIIA disease, all of which are good prognostic factors for stage III unresectable NSCLC. Furthermore, the OS Kaplan-Meier plots of the placebo groups revealed that the PD-L1-negative population overperformed the PD-L1-positive population (and, to a greater extent, the PD-L1>25% population) (Figure 1) [23–26]. Notably, the Kaplan-Meier plots were important for this interpretation, as the information could not be extrapolated from the 4-year HR values alone.

## Examples of Incomplete Reporting

The primary endpoint of the ExteNET trial was to determine whether neratinib treatment after standard adjuvant trastuzumab-containing therapy improves the 2-year invasive disease-free survival (IDFS) of patients with human epidermal growth factor receptor 2 (HER2)-positive early breast cancer. Adding neratinib did improve the 2-year IDFS from 91.6% to 93.9%, however, a higher incidence of grade 3/4 adverse events was noted [27]. Subsequently, several international guidelines recommended neratinib for select subgroups of patients. The EMA recommended neratinib for patients with hormone receptor-positive tumors within 1 year of trastuzumab-based therapy [28]. Analysis of this population was not a formal endpoint of this study, however, this EMA guidance is supported by the publication of Chan et al [29] which provided a complete dataset of clinical information on this group of interest. The NCCN guidelines recommended considering neratinib treatment for patients with hormone receptor-positive disease with lymph node involvement who underwent upfront surgery or did not achieve pathological complete response after neoadjuvant chemotherapy [30]. Analyzing these two subgroups were not defined as endpoints of the study, and the corresponding baseline patient and tumor characteristics were not included in the publications [27, 31]. Hence, it remains unclear whether within these subgroups, the treatment and placebo groups were well balanced, or whether imbalances that may have affected the results were in play.

The APHINITY trial assessed pertuzumab as an adjuvant treatment for HER2-positive early breast cancer. The primary endpoint was 3-year IDFS rate, which was 94.1% in the pertuzumab group and 93.2% in the placebo group (HR, 0.81; 95% CI, 0.66–1.00; *p* = 0.045). Subgroup analysis revealed that pertuzumab was more beneficial for select subgroups [32]. Consequently, the FDA, ESMO, and EMA recommended pertuzumab for high-risk patients, defined as patients with lymph node involvement or patients with hormone receptor-negative disease [33–35]. As in the ExteNET example, baseline patient and tumor characteristics for these subgroups were not reported [32].

The analysis of the APHINITY trial data emphasizes the challenges associated with interpreting statistically significant results from subgroup analyses. The original publication reported that the three subgroups benefiting the most from pertuzumab were postmenopausal patients, patients with node-positive disease, and those whose tumors were <2 cm in size. In a 2-variant analysis involving nodal status and tumor size, tumor size became a nonsignificant variable [32]. Interestingly, although nodal status was included in the guidelines as a decision-making factor for pertuzumab treatment, being postmenopausal was not, despite being a statistically significant predictor of treatment benefit (probably, because it was considered a type 1 error), further elucidating the need for careful examination of all subgroup analysis regardless of whether the findings are consistent with prior knowledge.

A more recent example of guidelines that were updated following an incomplete reporting of subgroup analyses involves treatment recommendations for young early-stage luminal breast cancer patients based on multigene expression assays. In the two phase III trials evaluating the 21-gene Recurrence Score (RS) assay in node-negative and node-positive hormone receptor-positive, HER2-negative breast cancer (TAILORx and RxPONDER, respectively), only younger patients (≤50 years in TAILORx, premenopausal in RxPONDER) benefited from adding chemotherapy to endocrine therapy (in TAILORx, the randomized arms included RS 11–25 patients and benefit was observed in the RS 16–25 range; in RxPONDER, the randomized arms included RS 0–25 patients, and the benefit was observed for the entire evaluated range) [36, 37]. Similarly, in an exploratory subgroup analysis of the phase III MINDACT trial evaluating the 70-gene signature in hormone receptor-positive, HER2-negative breast cancer, the chemotherapy benefit also seemed to be age-dependent with a clinically relevant effect observed only in those ≤50 years [38]. Notably, the published reports on TAILORx, RxPONDER, and MINDACT did not include baseline patient and tumor characteristics for the younger patient population by treatment arm, and the balance between the treatment arms within this subpopulation was not assessed [36–38]. Nonetheless, major treatment guidelines such as those published by ASCO and NCCN did incorporate these findings into their recommendations (the ESMO guidelines included these findings but did not provide specific recommendations) [30, 39, 40].

## Conclusion

The examples discussed in the current article illustrate the challenges associated with the interpretation of subgroup analyses. In order to address these challenges, and allow clinically meaningful interpretation that would ultimately improve patient care, we suggest that the standards for reporting results of subgroup analyses, particularly for subgroups of interest, should be the same as those for the main ITT population analysis; i.e., presenting Kaplan-Meier plots instead of just reporting the HR values, and including all patient/disease baseline characteristics for the subgroup of interest.

## Data Availability

The original contributions presented in the study are included in the article, further inquiries can be directed to the corresponding author.
